# Characteristics, Post-exposure prophylaxis usage, and clinical features of Chinese human rabies cases, 2016–2020

**DOI:** 10.1371/journal.pntd.0013089

**Published:** 2025-06-04

**Authors:** Qian Ren, Ning Chen, Di Mu, Man-Tong Zhu, Qing-Nan Shi, Shi-Jian Zhou, Hui-Jie Qin, Si-Han Li, Jun-Yuan Chen, Yu Li, Wen-Wu Yin, Qiu-Lan Chen, Yan-Ping Zhang

**Affiliations:** 1 National Key Laboratory of Intelligent Tracking and Forecasting for Infectious Diseases, Chinese Center for Disease Control and Prevention, Beijing, China; 2 Department of Comprehensive Management, Guangxi Academy of Medical Sciences and the People’s Hospital of Guangxi Zhuang Autonomous Region, Nanning, China; 3 School of Public Health, Guangxi Medical University, Nanning, China; 4 Beijing Children’s Hospital, Capital Medical University, National Center for Children’s Health, China; Universidade do Estado do Rio de Janeiro, BRAZIL

## Abstract

**Background:**

Rabies continues to pose a significant public health challenge in China. Here we reported the risk factors associated with the failure to initiate post-exposure prophylaxis (PEP) vaccination in China for period 2016–2020. It is essential for identifying strategies to decrease the incidence of these preventable fatalities.

**Methods:**

We analyzed data from 1,733 case investigations in China between 2016 and 2020. Chi-squared tests and logistic regression analyses were utilized to identify factors associated with the failure to initiate PEP vaccination.

**Results:**

A majority of the incidents (n = 1,295; 84.3%) involved animal bites, with dog wounds constituting 94.0% of these cases (n = 1,437). Domestic animals from the victims’ own residences accounted for 48.5% (n = 690) of human rabies cases, followed by free-roaming animals at 34.4% (n = 489). Among the domesticated animals, 95.7% (n = 779) had not been vaccinated against rabies. Category III wounds were present in 66.1% (n = 952) of all cases. In the multivariable model, factors occupation, exposure category, and source of attacking animal were significantly associated with failure to initiate PEP vaccination. However, only 4.3% (n = 41) received rabies immunoglobulin (RIG), and a mere 1.2% (n = 11) underwent complete PEP vaccination in addition to RIG administration. The median incubation period for rabies was recorded at 72 days, with an interquartile range (IQR) of 35.0–173.0 days.

**Conclusions:**

The majority of individuals affected by rabies did not pursue PEP vaccination, especially those with category II wounds or those bitten by domestic animals from their own households. To decrease mortality from rabies, it is imperative to launch prevention campaigns directed at these groups. Furthermore, enhancing the regular vaccination of domestic dogs is crucial for long-term control of the disease.

## Author summary

Rabies remains a significant public health challenge in China, with 202 human deaths reported in 2020. Domestic dogs are the primary source of human rabies cases, but vaccination rates among these animals are low. This study analyzes 1,733 human rabies case investigations in China from 2016-2020, providing insights into exposure history, post-exposure prophylaxis (PEP) use, and clinical characteristics. The majority of individuals affected by rabies did not pursue PEP, especially those with category II wounds or those bitten by domestic animals from their own households. Among the domestic animals involved, 95.7% were unvaccinated against rabies. The findings highlight the need for targeted educational initiatives on rabies prevention directed at high-risk groups like rural inhabitants, males, farmers, and older adults. Increasing accessibility and affordability of PEP for humans and enhancing management and vaccination of domestic dogs, especially in rural areas, are crucial for decreasing human rabies mortality. Achieving the goal of eliminating dog-mediated human rabies deaths by 2030 in China requires sustained, multisectoral commitment to the One Health approach.

## Background

Rabies is a lethal disease caused by the rabies virus [1–3]. It results in approximately 59,000 human fatalities globally each year, with around 40% of these deaths among individuals under 15 years of age and 95% in developing countries across Asia and Africa [4]. Uncontrolled populations of rabid animals can lead to outbreaks, posing a significant health security concern [5]. China, one of the countries most burdened by this disease, records 202 human deaths from rabies in 2020 [6].

Vaccination of dogs is recognized as the most cost-effective strategy for preventing human rabies [7]. However, vaccination rates are notably low due to factors such as geographical inaccessibility, limited awareness, the inconvenience of accessing vaccination services, a shortage of trained veterinarians, and a large population of free-roaming dogs, remaining well below the required 70% to halt transmission in canine populations [5,8].

The Chinese government has implemented robust measures to prevent and control rabies, which is a legally notifiable infectious disease in China. All clinical diagnoses and laboratory-confirmed cases of rabies must be reported to the National Notifiable Infectious Disease Reporting Information System (NNIDRIS). In alignment with the World Health Organization’s Expert Consultation on Rabies technical standards (2018 version) [9], China has developed protocols and technical guidelines for post-exposure prophylaxis (PEP) following animal injuries. Both category II and III exposures require wound treatment and PEP vaccination. Additionally, rabies immunoglobulin (RIG) must be administered for category III exposures. The PEP vaccination can be conducted using the Zagreb 2–1–1 regimen, which involves administering two vaccine doses intramuscularly on day 0 (one in each deltoid or thigh) followed by a single dose on days 7 and 21, or the five-dose Essen regimen, with one dose given intramuscularly on days 0, 3, 7, 14, and 28, as recommended by the World Health Organization (WHO). It is crucial that patients with category II or III exposures begin their vaccination series promptly after potential exposure to rabies [5].

Despite significant advancements in treatment over the past decade, the incidence of rabies decreased significantly, with 202 reported cases across 143 counties and districts in 2020, reflecting a 94% and 86% reduction from the peak in 2007, when there were 3,300 cases across 984 counties and districts, rabies continues to pose a severe health risk in China [6,10]. Recent data indicate that rabies consistently ranks among the top five most deadly infectious diseases in the country, representing a substantial threat to public health [7]. In this study, we analyzed human rabies case investigations conducted from 2016 to 2020 to describe the exposure history, clinical characteristics, and PEP practices in China. Our findings aim to inform future interventions, enhance community awareness, and improve clinical practices concerning rabies exposure.

## Materials and methods

1. Ethics Statement: Data for this study were derived from continual public health monitoring of a designated notifiable infectious disease. The National Health and Family Planning Commission of China classified the investigation of human rabies incidents as a perpetual public health surveillance effort, exempting it from institutional ethical review board evaluation. Similarly, this project was recognized as a standard public health surveillance activity, aligning with the human subjects’ protection protocols of the United States CDC (CGH #2015–238). All data used in the analysis were anonymized, containing no personally identifiable information. All investigations were conducted with verbal informed consent from the patient’s family.
2. Study design: A cross-sectional survey was used to investigate all cases of rabies diagnosed in 2016–2020. Considering that death occurs within 1 week after the onset of this disease, face-to-face surveys of patients’ family members were used, with Center for Disease Control and Prevention (CDC) personnel from the affiliated districts and counties serving as investigators. The Chinese CDC designed a uniform questionnaire and trained the investigators in a uniform manner.
3. Data source: The dataset was derived from a questionnaire survey covering 31 provinces in China, excluding Hong Kong S.A.R., China, Macau S.A.R., China, Taiwan, China, and international cases. It includes case investigation results spanning from January 1, 2016, to December 31, 2020.
4. Content of the survey: Includes demographic and sociologic characteristics, exposure and wound management, post-exposure prophylaxis, clinical information, laboratory testing, injured animals, and so on.
5. Methods to address potential bias: Epidemiologic investigations are conducted immediately after a rabies case is reported to CDC to minimize recall bias and missing data.
6. Case definition: This analysis includes all cases that conform to the classifications of “clinically diagnosed cases” and “confirmed cases” as defined by the People’s Republic of China health industry standard “Diagnostic Criteria for Rabies” (WS281–2008) [11]. “Suspected cases” are excluded from this analysis.
  Cases clinically diagnosed are consistent with the symptoms of manic rabies or display clinical signs of paralytic rabies accompanied by a known epidemiological history of rabies exposure.
  Confirmed cases: Confirmed cases include clinically diagnosed incidents where antibodies were detected using direct fluorescent antibody (DFA) or enzyme-linked immunosorbent assay (ELISA), nucleic acids were identified through RT-PCR, or the rabies virus was isolated in tissue samples via cell culture.
7. Analytical approach: This study seeks to identify the factors contributing to rabies infections following animal injuries by conducting an epidemiological analysis of exposure history, wound management, and PEP practices. The findings will inform future interventions, identify high-risk groups and conduct educational actions with the population, enhance community awareness, improve clinical practices concerning rabies exposure, and inform the development of targeted rabies prevention and control strategies and support the creation of policy frameworks aimed at achieving the rabies elimination goal by 2030.
8. Statistical analysis: Data were organized, analyzed, and graphically represented using Microsoft Excel 2016. The demographic characteristics of human rabies cases were described using both Excel 2016 and SPSS V28. Categorical variables were analyzed using percentages for descriptive purposes. The Chi-squared Test and logistic regression were utilized to determine factors associated with the non-initiation of PEP vaccination. In the logistic regression analysis, univariate and multivariate analyses were performed to explore the independent effect of each independent variable on the dependent variable and the effect of each independent variable on the dependent variable after adjusting for other variables. In multivariable logistic regression analyses, we checked for the presence of multicollinearity among independent variables, and the way variables entered the model was “enter”. *P*-values less than 0.05 were deemed statistically significant.

## Results

### 1. Characteristics of cases and rabies exposures

We gathered data from 1,733 case investigations spanning from January 1, 2016, to December 31, 2020, across 23 of the 31 provincial-level administrative regions in China. Notably, no rabies cases were reported from Beijing, Zhejiang, Qinghai, Inner Mongolia, Heilongjiang, Jilin, Tibet, and Liaoning during the study period.

Of the 1,733 rabies cases, 1,538 (88.7%) were clinically diagnosed, while 195 (11.3%) were confirmed through laboratory testing. Most cases occurred in rural areas (n = 1,519; 93.2%), predominantly among males (n = 1,212; 70.1%) and farmers (n = 1,271; 75.6%). The majority of the patients were aged 65 years or older (n = 526; 30.5%), followed by age groups 55–64 years (n = 370; 21.5%), 45–54 years (n = 328; 19.0%), and those younger than 15 years (n = 228; 13.2%). The median age was 56.0 years, with an interquartile range (IQR) of 41.0-66.0 years.

The most common exposure route was animal bites, accounting for 84.3% (n = 1,295) of cases (Table 1). Of these, wounds from dogs and cats constituted 94.0% (n = 1,437) and 5.0% (n = 77), respectively. The majority of human rabies cases originated from domesticated animals in the victim’s own home (n = 690; 48.5%), free-roaming animals (n = 489; 34.4%), and domesticated animals from the neighborhood (n = 208; 14.6%). Among the domesticated animals involved, 95.7% (n = 779) had not been vaccinated against rabies. Aggressive behavior was noted as the cause in more than half of the instances (n = 817; 55.8%), and in some cases, the same animal attacked more than one individual (n = 178; 12.6%).

**Table 1:** Characteristics of exposure history and post-exposure prophylaxis, according to human rabies case investigations data, China, 2016–2020.

Characteristics	Diagnosis type, n (%)		Total, n (%)
	Clinically diagnosed cases	Laboratory-confirmed cases	
**Source of attacking animal**			
Domesticated animals from own home	613 (48.4)	77 (49.7)	690 (48.5)
Domesticated animals from the neighborhood	181 (14.3)	27 (17.4)	208 (14.6)
Free roaming animal	442 (34.9)	47 (30.3)	489 (34.4)
Wild animal	15 (1.2)	3 (1.9)	18 (1.3)
Others	16 (1.3)	1 (0.6)	17 (1.2)
Total	1267 (100.0)	155 (100.0)	1422 (100.0)
**Exposure category**			
II	320 (24.9)	39 (24.8)	359 (24.9)
III	853 (66.5)	99 (63.1)	952 (66.1)
II or III^A^	97 (7.6)	12 (7.6)	109 (7.6)
Unknown^B^	13 (1.0)	7 (4.5)	20 (1.4)
Total	1283 (100.0)	157 (100.0)	1440 (100.0)
**Type of wound treatment**			
No treatment	782 (60.1)	99 (61.9)	881 (60.3)
Treated by oneself	408 (31.3)	50 (31.3)	458 (31.3)
Treated in a medical institution	112 (8.6)	11 (6.9)	123 (8.4)
Total	1302 (100.0)	160 (100.0)	1462 (100.0)
**Receive PEP vaccination** ^C^			
Yes^D^	115 (9.8)	10 (7.5)	125 (9.6)
No	1053 (90.2)	123 (92.5)	1176 (90.4)
Total	1168 (100.0)	133 (100.0)	1301 (100.0)
**Injecting RIG or not**			
Yes	46 (3.9)	5 (3.5)	51 (3.8)
No	1136 (96.1)	139 (96.5)	1275 (96.2)
Total	1182 (100.0)	144 (100.0)	1326 (100.0)

Note: ^A^ Exposure caused by an animal bite or scratch but classified as category I by medical staff.

^B^Exposure was classified as category I by medical staff, but the exposure route was unknown.

^C^Received at least one dose of rabies vaccine.

^D^69 (55.2%) cases received Vero cell rabies vaccine, and 7 (5.6%) received hamster kidney cell rabies vaccine. 49 cases (39.2%) were missing data.

Our analysis identified that 129 cases involved category I wounds. Upon verifying exposure routes, it was determined that 109 of these cases resulted from animal bites or scratches. The lack of comprehensive detail on the wounds, however, prevented definitive categorization, leading to their reclassification as category “II or III” wounds. The remaining 20 cases were designated as “unknown” wound categories due to incomplete data or indeterminate exposure routes. Notably, the majority of cases were classified under “category II” (24.9%, n = 359), “category III” (66.1%, n = 952), “category II or III” (7.6%, n = 109), or “unknown” (1.4%, n = 20). A significant number of human rabies cases involved bites to highly innervated regions, including the hand (56.9%, n = 624), neck (2.5%, n = 23), and head and face (15.8%, n = 151).

### 2. Wound treatment, PEP vaccination, and RIG

The analysis of case investigation data revealed that only 123 cases (8.4%) underwent wound treatment at medical institutions, of which 74 cases received appropriate treatment, including wound flushing and disinfection. Conversely, 881 cases (60.3%) did not receive appropriate wound care. Furthermore, 125 cases (9.6%) commenced a PEP vaccination regimen against rabies. Of these, 87 cases (69.6%) started the five-dose Essen regimen, 8 cases (6.4%) the 2-1-1 Zagreb regimen, and 30 cases (24%) did not have a specified vaccination schedule. Among the 51 cases (3.8%) that received RIG, 37 cases (72.5%) were administered human rabies immune globulin (HRIG), while the remaining 14 cases (27.5%) received equine rabies antiserum (ERA).

In the bivariate analysis, no statistically significant difference was observed between the sexes in initiating PEP vaccination (P = 0.484). However, statistically significant associations were found with several factors: occupation (P < 0.001), age (P = 0.004), area of residence (P = 0.032), exposure category (P < 0.001), and the source of the attacking animal (P = 0.001), all influencing the failure to initiate PEP vaccination.

In the multivariable model, no multicollinearity among independent variables. Factors occupation, exposure category, and source of attacking animal were significantly associated with failure to initiate PEP vaccination. Children are more likely to begin PEP vaccination compared to adults (aOR = 8.182, 95% CI: 1.442-46.440). Similarly, those exposure categories III are more likely to begin PEP vaccination compared to those other exposure categories (aOR = 4.924, 95% CI: 2.224-10.904), and those attacks from “domesticated animals from the neighborhood” had an aOR of 2.691 (95% CI: 1.438-5.035), and attacks from “free roaming animals” had an aOR of 1.969 (95% CI: 1.157-3.351). (Table 2).

**Table 2:** Risk factors associated with failure to begin PEP vaccination, according to data obtained from human rabies case investigations, China, 2016–2020 (N = 1311*).

Factor	Cases beginning vaccination (%)	Cases NOT beginning PEP vaccination (%)	Adjusted OR (95% CI)
**Sex**			
Female	39 (34.5)	304 (31.3)	Ref
Male	74 (65.5)	668 (68.7)	0.892 (0.545-1.460)
Total	113 (100.0)	972(100.0)	
**Occupation**			
Others^A^	11 (10.2)	70 (7.4)	Ref
Farmer	77 (71.3)	785 (82.8)	1.041 (0.407-2.664)
Student^B^	9 (8.3)	72 (7.6)	1.788 (0.404-7.921)
Children^C^	11 (10.2)	21 (2.2)	**8.182 (1.442-46.440)**
Total	108 (100.0)	948 (100.0)	
**Age (years)**			
<15	21 (18.6)	89 (9.2)	Ref
15-54	43(38.1)	356(36.7)	1.378 (0.323-5.882)
≥55	49 (43.4)	525 (54.1)	1.155 (0.270-4.943)
Total	113 (100.0)	970 (100.0)	
**Area**			
Urban	11 (10.5)	50 (5.3)	Ref
Rural	94 (89.5)	892 (94.7)	0.614 (0.248-1.521)
Total	105 (100.0)	942 (100.0)	
**Exposure category**			
II	11 (9.7)	276 (28.3)	Ref
III	102 (90.3)	699 (71.7)	**4.924 (2.224-10.904)**
Total	113 (100.0)	975 (100.0)	
**Source of attacking animal**			
Domesticated animals from own home	30 (29.7)	448 (49.0)	Ref
Domesticated animals from the neighborhood	25 (24.8)	129 (14.1)	**2.691 (1.438-5.035)**
Free roaming animals	45 (44.6)	317 (34.7)	**1.969 (1.157-3.351)**
Wild animal	1 (1.0)	9 (1.0)	2.315 (0.257-20.853)
Others	–	11 (1.2)	0.000 (0.000)
Total	101 (100.0)	914 (100.0)	

Note: *422 cases were not included, of which 293 cases didn’t answer what exposure category and 129 cases belonged to the I exposure category.

OR = odds ratio. Ref = reference.

^A^Workers, laborers, self-employed and unemployed, workers, food industry personnel, retired and cadres of staff, etc.

^B^Primary, secondary, and college students.

^C^Children attending or not attending kindergarten.

Among the individuals who initiated PEP following category II or higher exposures (n = 118), only 24 (20.3%) completed the full vaccination series as per the prescribed schedule—22 adhered to the five-dose Essen regimen and 2 followed the 2–1–1 Zagreb regimen (Table 3). Of the 952 individuals with category III exposures, 41 (4.3%) received RIG and a mere 11 (1.2%) completed both the PEP vaccination series and RIG administration. Among the 101 individuals who commenced but did not complete the PEP vaccination series, 53 (52.5%) developed rabies symptoms during the vaccination schedule. Additionally, 7 individuals (6.9%) believed it was unnecessary to complete the series, 3 (3.0%) experienced physical discomfort, and 1 (1.0%) could not afford the remaining vaccinations. The reasons were unreported for the remaining 37 cases (36.6%) with incomplete vaccination series.

**Table 3:** Completion of post-exposure prophylaxis by wound exposure category, according to.

Item	Exposure category, % (n)			Total, % (n)
	II	III	II or IIIc	
Wound treatment^A^	3.9% (14/359)	10.3% (98/952)	5.5% (6/109)	8.3% (118/1420)
Vaccination^B^	0.6% (2/359)	2.2% (21/952)	0.9% (1/109)	1.7% (24/1420)
anti-rabies serum	1.9% (7/359)	4.3% (41/952)	0.9% (1/109)	3.5% (49/1420)
Wound treatment^A^+Vaccination^B^	0.3% (1/359)	1.8% (17/952)	0.9% (1/109)	1.3% (19/1420)
Wound treatment^A^+Vaccination^B^+ anti-rabies serum	0.3% (1/359)	0.9% (9/952)	0.0% (0/109)	0.7% (10/1420)

Note:^A^ Normative wound treatment in a medical institution.

^B^Complete vaccination course.

^C^Exposure caused by animal bitten or scratched but classified as category I by medical staff.

data obtained from human rabies case investigations, China, 2016–2020.

Among the 53 individuals who exhibited rabies symptoms prior to completing their vaccination regimen, 11 (20.8%) did not receive the recommended wound treatment at a healthcare facility, and 20 (37.7%) who had category III exposure and bites in areas with dense nerve supply (such as the head, face, neck, and hands) did not receive RIG. Furthermore, of these 53 cases, 34 (64.2%) received medical treatment for their wounds at a facility within a day of exposure, 37 (69.8%) initiated PEP vaccination within one day, but only 18 (34.0%) received RIG within this timeframe. Regarding outcomes, 45 (84.9%) progressed from exposure to morbidity within one month and 46 (86.8%) from morbidity to death within one week.

The majority of individuals classified with category II or higher exposure received wound treatment (n = 485; 96.0%), PEP vaccination (n = 80; 73.4%), and RIG (n = 32; 74.4%) at a medical facility on the same day of exposure (Table 4).

**Table 4:** Time interval from rabies exposure to post-exposure prophylaxis by exposure.

Interval	Exposure category, n (%)			Total, n (%)
	II	III	II or III^A^	
**From exposure to wound treatment**				
Within 1 day	86 (98.9)	364 (95.5)	35 (94.6)	485 (96.0)
1 day	1 (1.1)	9 (2.4)	0 (0.0)	10 (2.0)
> 1 day	–	8 (2.1)	2 (5.4)	10 (2.0)
Total	87 (100.0)	381 (100.0)	37 (100.0)	505 (100.0)
**From exposure to vaccination**				
Within 1 day	7 (77.8)	71 (73.2)	2 (66.7)	80 (73.4)
1 day	1 (11.1)	10 (10.3)	1 (33.3)	12 (11.0)
> 1 day	1 (11.1)	16 (16.5)	0 (0.0)	17 (15.6)
Total	9 (100.0)	97 (100.0)	3 (100.0)	109 (100.0)
**From exposure to injecting RIG**				
Within 1 day	2 (50.0)	30 (76.9)	–	32 (74.4)
1 day	1 (25.0)	5 (12.8)	–	6 (14.0)
> 1 day	1 (25.0)	4 (10.3)	–	5 (11.6)
Total	4 (100.0)	39 (100.0)	–	43 (100.0)
**From morbidity to death**				
≤7 days	291 (87.4)	821 (91.8)	86 (87.8)	1198 (90.4)
8 days - 1 month	38 (11.4)	64 (7.2)	12 (12.2)	114 (8.6)
1 month – 3 months	3 (0.9)	6 (0.7)	0 (0.0)	9 (0.7)
≥3 months	1 (0.3)	3 (0.3)	0 (0.0)	4 (0.3)
Total	333 (100.0)	894 (100.0)	98 (100.0)	1325 (100.0)

Note:

^A^Exposure caused by animal bitten or scratch but classified as category I by medical staff.

category, according to data obtained from human rabies case investigations, China, 2016–2020.

### 3. Clinical characteristics of the rabies cases

In this study, we analyzed the clinical characteristics of 1,542 rabies cases. The majority of cases exhibited severe manifestations: hydrophobia was reported in 1,256 cases (81.5%), agitation in 1,242 cases (80.5%), and aerophobia in 1,180 cases (76.5%). Additionally, 27.4% of cases displayed mental disorder symptoms (Table 5). Both clinically diagnosed and laboratory-confirmed cases presented similar clinical manifestations.

**Table 5:** Clinical manifestations of human rabies cases by diagnosis type, China, 2016–2020.

Manifestation	All cases (N = 1542)		Clinically diagnosed cases		Laboratory-confirmed cases	
	Cases (n)	%	Cases (n)	%	Cases (n)	%
Agitation	1242	80.5	1101	80.4	141	81.5
Hydrophobia	1256	81.5	1122	82.0	134	77.5
Aerophobia	1180	76.5	1046	76.4	134	77.5
Photophobia	819	53.1	740	54.1	79	45.7
Convulsions	632	41.0	572	41.8	60	34.7
Mental disorder^A^	422	27.4	375	27.4	47	27.2
Total	1542	100.0	1369	100.0	173	100.0

^A^Mental status alternates between an established normal baseline and progressively more severe agitation and/or depression.

The median incubation period, defined as the time from exposure to symptom onset, was 72 days overall (IQR 35.0–173.0). For category II, the median incubation period extended to 89 days (IQR 38.0–240.0), while it was 67 days (IQR 33.5–155.5) for category III. Children under 15 years had the shortest median incubation period across all age groups, recorded at 54 days (IQR 20–184). The median duration of the clinical course, from symptom onset to death, spanned 3 days (IQR 2–5) for all cases.

## Discussion

Over the past decade, substantial governmental efforts have been directed towards rabies prevention and control. In 2020, the incidence of rabies decreased significantly, with 202 reported cases across 143 counties and districts, reflecting a 94% and 86% reduction from the peak in 2007, when there were 3,300 cases across 984 counties and districts [6]. Despite these achievements, China continues to face one of the highest rabies burdens globally. This national-level analysis aims to enrich our understanding of human rabies through detailed case investigations, highlighting exposure history, PEP usage, and clinical features of the disease in China. The study reveals that the majority of infections occur predominantly among males, farmers, and individuals aged middle-aged and older, primarily residing in rural areas. These findings are consistent with those reported in several other studies [5,12]. The high proportion of rabies in rural areas may be due to a lack of awareness and limited accessibility to PEP owing to economic constraints or geographical distance [13].

In China, pre-exposure prophylaxis vaccination rates for rabies remain critically low among the general population [5]. Similarly, vaccination rates among domestic animals are deficient; in the study, 95.7% (779 animals) were unvaccinated against rabies. Consequently, rabies prevention predominantly depends on PEP, which involves proper wound care, completion of the rabies PEP vaccination schedule, and the use of RIG when necessary [9,14]. Findings indicate that most human rabies cases, including those with category III exposure, did not receive standard wound care or PEP vaccination at healthcare facilities.

Appropriate and timely wound treatment can diminish rabies viral loads and the risk of secondary bacterial infections at bite or scratch sites [5]. Nevertheless, in our study, only 74 (5.1%) cases received proper wound care, including flushing and disinfection, at the medical facility.

In our study, a small percentage of human rabies cases initiated the PEP series, a finding consistent with data from other countries [15,16]. PEP for rabies, while generally effective, occasionally fails, with reports of sporadic breakthrough infections. Ensuring timely and proper administration of PEP is vital for preventing rabies. Even with strict adherence to recommended procedures, individuals with high-risk exposures or immunocompromised states may rarely contract rabies [17]. Incomplete PEP schedules reduce the likelihood of developing protective antibody titers necessary for infection prevention. Our analysis highlights that both the initiation and completion rates of the full PEP vaccination series were low, significantly influencing the incidence of rabies. Specifically, only 9.6% of human rabies cases began the PEP series, and fewer than a quarter of these completed the required vaccinations. Predominantly, individuals who contracted rabies did not seek PEP. This trend was especially pronounced among those with category II wounds or those bitten by domestic animals from their own households.

For category III exposure cases, the administration of RIG is recommended along with wound treatment and PEP vaccination [18]. However, our study found that a small percentage of these cases received the appropriate RIG treatment, indicating that the failure to administer RIG is a significant risk factor for the development of rabies.

The adherence to recommended protocols for PEP may be hindered by several factors, including the substantial out-of-pocket expenses for PEP vaccines and RIG, limited awareness regarding rabies prevention, and inadequate PEP practices within medical facilities. Enhancing compliance with PEP guidelines in healthcare institutions is crucial and should be a focal point of rabies intervention campaigns. The average disposable income for rural communities in China was 17,131 CNY (about 2,347 USD) in 2020 [6]. The costs for a full course of PEP vaccination and RIG are approximately 300 CNY (about 41 USD) and 1,000 CNY (about 137 USD) respectively [5]. Additional financial burdens include the costs associated with wound care, transportation, and income loss due to treatment. These costs impose significant economic strain on low-income individuals. It is advisable to consider increasing the proportion of PEP expenses covered by Medicare. Study findings indicate that rural inhabitants, males, farmers, and older adults are particularly vulnerable to rabies exposure. Consequently, targeted educational initiatives focusing on rabies awareness, bite prevention, and infection control are imperative for these high-risk groups. Furthermore, healthcare facilities should conduct regular reviews of their rabies PEP protocols to enhance practitioner competence and support the success of future rabies prevention efforts.

Among individuals with category II or higher exposure levels who completed the full PEP vaccination series (n = 24), the five-dose Essen regimen was administered to 22 (91.7%) cases, while the 2–1–1 Zagreb regimen was used in 2 (8.3%) cases. We recommend broader implementation of the Zagreb regimen or the 1-1-1-1 regimen (a single intramuscular injection administered on days 0, 3, 7, and between days 14–28) in China [18]. The reduced number of clinic visits associated with these regimens can enhance PEP vaccination compliance by decreasing both direct vaccination and travel expenses as well as minimizing loss of work time.

According to the WHO and Chinese national guidelines, category I exposures are not associated with active rabies infections [5,18,19]. Our original dataset included 129 cases initially classified by healthcare professionals as category I exposures. However, following a review based on the nature of the exposures and adherence to our standardized classification system for rabies, 109 (84.5%) of these cases were reclassified as either category II or III exposures. This discrepancy highlights the need for enhanced training and standardization in the categorization of rabies exposures in PEP clinics. Accurate rabies burden assessment requires the improvement of laboratory confirmation rates for suspected human rabies cases.

Category III exposures, characterized by large and deep wounds, resulted in shorter incubation periods due to high viral inoculation loads [20]. Children under 15 years exhibited shorter incubation periods, potentially because their frequent close interactions with pets, such as dogs and cats, often lead to bites in highly enervated areas like the head, face, neck, and hands [20,21]. Consequently, implementing infection prevention education in schools is advised.

In our previous study, we conducted a Stepwise Approach towards Rabies Elimination (SARE) evaluation that identified key activities for eliminating dog-induced rabies as including improved surveillance of dog rabies and increased dog vaccination coverage [22]. In our another study, based on data from China’s national human rabies surveillance system, we used decision-analytic modelling to estimate dog-mediated human rabies death trends in China, suggest that increased integrated bite case management (IBCM) could reduce unnecessary PEP use and make the One Health rabies control strategy most cost-effective [23]. In this study, we identified dogs as the primary source of human rabies, with most cases stemming from domesticated animals from their own home. Similar findings were reported in other countries including India [15], Pakistan [24], and Indonesia [25]. Domestic dogs present the highest risk to global public health among rabies reservoir species [26], suggesting possible interactions between domesticated dogs and wildlife. Among the examined domestic animals, 779 (95.7%) were not vaccinated against rabies, and 178 (12.6%) had attacked multiple individuals. Vaccination of dogs is recognized as the most cost-effective strategy for preventing human rabies [7]. However, vaccination rates are notably low due to factors such as limited awareness, the inconvenience of accessing vaccination services, a shortage of trained veterinarians, and a large population of free-roaming dogs, remaining well below the required 70% to halt transmission in canine populations [5]. To address this, there is a need to strengthen management and prioritize canine immunity [27]. China has adopted the goal to eliminate rabies transmitted by dogs in the coming years, a challenge that necessitates nationwide compulsory vaccination enacted through a multisectoral approach under the “One Health” framework [28]. The 2021 revision of the Animal Epidemic Prevention Law of the People’s Republic of China [29] mandates that dog owners vaccinate their pets against rabies, providing a legal framework to support the eradication efforts [6]. The cost for annual dog registration, which includes rabies vaccination, varies across provinces, ranging from $28 to $140 [22]. Proposed measures to enhance vaccination accessibility in rural areas include establishing more local vaccination sites, increasing mobile, and possibly implementing door-to-door vaccination services. Additionally, improving compensation and subsidies for veterinarians, expanding the veterinary workforce in rural regions, diversifying vaccine options available through free programs, and intensifying public awareness campaigns about dog vaccination strategies are crucial steps [30].

Our study had some limitations. Considering that death occurs within 1 week after the onset of this disease, face-to-face surveys of patients’ family members were used. Epidemiologic investigations are conducted immediately after a rabies case is reported to CDC to minimize recall bias. But there is still some missing data. Therefore, we were not able to assess whether the exposure characteristics and clinical features of the cases included in this analysis were representative of all cases. Nevertheless, our large sample size and analysis provide important information for rabies control in China.

The WHO, the World Organization for Animal Health (WOAH), the Food and Agriculture Organization (FAO), and the Global Alliance for Rabies Control (GARC) have set a goal to eliminate dog-mediated rabies by 2030 [22]. Numerous countries have successfully eliminated rabies through both human and domestic dog vaccinations using the “One Health” approach [23]. The WHO and its partners aim to achieve the elimination of dog-mediated human rabies deaths in up to 92% of countries worldwide by 2030, a target known as “Zero-by-30,” while all countries should reduce such deaths by 50% [31,32]. In China, the government’s adoption of a multi-sectoral “One Health” strategy has significantly advanced the fight against rabies in recent years [33–35].

## Conclusions

The goal of “Zero rabies deaths by 2030” may be achieved by establishing enduring herd immunity in dogs, implementing cost-effective human PEP, and enhancing community education. Furthermore, comprehensive monitoring and reporting of all cases are essential for precise prevention and control strategies. Additionally, the development of vaccines appropriate for stray cats and dogs in both urban and rural settings, as well as for foxes, wolves, raccoons, and other wildlife, needs to be intensified.
